# Ginsenoside ameliorated ventilator-induced lung injury in rats

**DOI:** 10.1186/s40560-020-00509-5

**Published:** 2020-11-23

**Authors:** Woo Hyun Cho, Yun Hak Kim, Hye Jin Heo, Dohyung Kim, Tae Won Kwak, Kwang Ho Kim, Hye Ju Yeo

**Affiliations:** 1 Division of Pulmonary, Allergy and Critical Care Medicine, Department of Internal Medicine, Research Institute for Convergence of Biomedical Science and Technology, Pusan National University Yangsan Hospital, Geumo-ro 20, Beomeo-ri, Mulgeum-eup, Yangsan-si, Gyeongsangnam-do 626-770 Republic of Korea; 2 Department of Anatomy and Department of Biomedical Informatics, School of Medicine, Pusan National University, Busan, Republic of Korea; 3 Department of Thoracic and Cardiovascular surgery, Pusan National University Yangsan Hospital, Yangsan-si, Republic of Korea; 4 Medical Convergence Materials Commercialization Center, GyeongBuk TechnoPark, Gyeongbuk, Republic of Korea

**Keywords:** Ginsenosides, Ventilator-induced lung injury, Tumor necrosis factor-alpha, LCN2, NGAL

## Abstract

**Background:**

Ginsenosides have antioxidant and anti-inflammatory features. This study aimed to evaluate the biologic effects of ginsenoside Rb2 pretreatment on ventilator-induced lung injury (VILI) in rats.

**Methods:**

Rats were divided into four groups with 12 rats per group: control; low tidal volume (TV), TV of 6 mL/kg, VILI, TV of 20 mL/kg, positive end-expiratory pressure of 5 cm H_2_O, and respiratory rate of 60 breaths per minute for 3 h at an inspiratory oxygen fraction of 0.21; and ginsenosides, treated the same as the VILI group but with 20 mg/kg intraperitoneal ginsenoside pretreatment. Morphology was observed with a microscope to confirm the VILI model. Wet-to-dry weight ratios, protein concentrations, and pro-inflammatory cytokines in the bronchoalveolar lavage fluid were measured. RNA sequencing of the lung tissues was conducted to analyze gene expression.

**Results:**

High TV histologically induced VILI with alveolar edema and infiltration of inflammatory cells. Ginsenosides pretreatment significantly reduced the histologic lung injury score compared to the VILI group. Wet-to-dry weight ratios, malondialdehyde, and TNF-α in bronchoalveolar lavage fluid were significantly higher in the VILI group and ginsenoside pretreatment mitigated these effects. In the immunohistochemistry assay, ginsenoside pretreatment attenuated the TNF-α upregulation induced by VILI. We identified 823 genes differentially presented in the VILI group compared to the control group. Of the 823 genes, only 13 genes (*Arrdc2*, *Cygb*, *Exnef*, *Lcn2*, *Mroh7*, *Nsf*, *Rexo2*, *Srp9*, *Tead3*, *Ephb6*, *Mvd*, *Sytl4*, and *Ube2l6*) recovered to control levels in the ginsenoside group.

**Conclusions:**

Ginsenosides inhibited the inflammatory and oxidative stress response in VILI. Further studies are required on the 13 genes, including LCN2.

## Background

Acute lung injury (ALI) and acute respiratory distress syndrome (ARDS) are fatal diseases [1]. Mechanical ventilation is essential for overcoming life-threatening hypoxia and hypercapnia. Despite the lifesaving effects, mechanical ventilation may induce or deteriorate lung injury, a state noted as ventilator-induced lung injury (VILI) [2]. VILI involves neutrophil activation, high permeability pulmonary edema, increased vascular permeability, and inflammation [2]. Hyperinflated lung caused by mechanical stretch induces a biochemical response, which facilitates neutrophils and inflammatory signals [3]. Increased inflammatory mediators such as cytokines cause complex interactions [4]. The potential mechanisms regarding the transition from mechanical stress to a biochemical reaction have been widely evaluated. Despite the intense interest in a therapeutic target for treating VILI, no available pharmacologic option exists.

Panax ginseng is the most commonly used herbal medicine since ancient times. Most of the biological functions of Panax ginseng come from ginsenosides [5]. Ginsenosides have anti-inflammatory activities achieved by inhibiting the production of pro-inflammatory cytokines and inhibiting the actions of inflammatory signaling pathways [6, 7]. In particular, ginsenoside Rb2 (GnRb2) significantly inhibits the transcriptional activity of NF-kB [8, 9]. Multiple studies have been conducted to understand the role and mechanisms of ginsenosides in a variety of diseases, including diabetes mellitus, cardiovascular disease, and cancers [10–13]. However, no related study has showed the effects of GnRb2 pretreatment on VILI. Considering the anti-inflammatory and antioxidant function, we hypothesized that GnRb2 will have protective effects in VILI. To verify this hypothesis, we ventilated rats with high tidal volume (TV) and evaluated the effects of GnRb2 in attenuating VILI in rats. Furthermore, we conducted a gene expression profiling analysis of the lung tissues to understand the underlying mechanism and identify potential therapeutic targets.

## Methods

### Materials

Korean red ginseng extract was manufactured by the Korea Ginseng Corporation (Seoul, Korea). GnRb2 was purified as described by Kitagawa et al. [14], and the purity was higher than 99.9%.

### Animal preparation

Eight-week-old Sprague-Dawley rats weighing 250–300 g were used. All surgeries were performed under ketamine anesthesia given intraperitoneally (100 mg/kg; Eurovet Animal Health BV, Bladel, the Netherland). The rats were randomly divided into four groups (*N* = 12 per group): control, low TV, VILI, and GnRb2. The control group underwent tracheostomy after anesthesia and breathed spontaneously without mechanical ventilation (MV). The low TV group was ventilated with an animal ventilator (model 683) (Harvard Apparatus, Holliston, MA, USA) using a TV of 6 mL/kg, positive end-expiratory pressure of 5 cmH_2_O, and respiratory rate of 60 breaths per minute for 3 h at an inspiratory oxygen fraction of 0.21. The VILI group was ventilated with an animal ventilator (model 683) (Harvard Apparatus, Holliston, MA, USA) using a tidal volume of 20 mL/kg. The VILI group has used the ventilator the same way as the low TV group except for high TV. The GnRb2 group was treated with 20 mg/kg GnRb2 intraperitoneally 5 h before ventilator initiation. And the GnRb2 group was treated the same as the VILI group. All rats were sacrificed 3 h after tracheostomy.

### Morphologic changes in the lung tissues

For histological evaluation, the lung specimen was fixed with the same condition with the lung expanded. Histological examination was performed on the right upper lobe of the lung as described in previous studies [15, 16]. Five non-overlapping fields of lung sections were examined by two pathologists (Knotus Co. Ltd., Incheon, Republic of Korea) who were blinded to the group of the rat. Lung injury was evaluated as described in [15] for (I) alveolar capillary congestion; (II) hemorrhage; (III) infiltration of neutrophils into the airspace, vessel wall, or alveolar wall; and (IV) alveolar wall thickness/hyaline membrane formation. Severity were scored as follows: 0 (normal), 1 (mild, < 25%), 2 (moderate, 25–50%), 3 (severe, 50–75%), 4 (very severe, > 75%). The lung injury score is the sum of all scores for each item.

### Assess of pulmonary edema

We evaluated pulmonary edema using wet-to-dry weight ratios. We sacrificed rats by arterial bleeding after 3 h ventilation and removed the entire lungs (six rats in each group). The lungs were weighed immediately (wet weight) and then placed in an oven at 60 °C for 72 h. After drying, the lungs were weighed again (dry weight). The wet-to-dry weight ratio was defined as the (wet weight-dry weight)/dry weight.

### Measuring protein concentrations and cytokines

Bronchoalveolar lavage fluid (BALF) was acquired from the left upper lung as described previously [16]. Myeloperoxidase (MPO) and malondialdehyde (MDA) in BALF were measured in a blinded fashion using the appropriate MPO (ab105136, Abcam, Cambridge, UK) or MDA (ab118970, Abcam, Cambridge, UK) kits (Supplement 1). Cytokines were measured in a blinded fashion, using rat-specific ELISA kits (Supplement 1).

### Immunohistochemical staining of TNF-α

TNF-α staining was performed on serial sections from six specimens per group. Tissues were incubated overnight at 4 °C with a rabbit polyclonal antibody against TNF-a (Abcam, Cambridge, UK). Primary antibody incubation was followed by the addition of donkey anti-rabbit IgG antibody incubation (Abcam, Cambridge, UK).

### RNA sequencing

We extracted RNA using formalin-fixed, paraffin-embedded (FFPE) lung tissues. RNA purity was controlled by analyzing 1 μL of total RNA extract on a NanoDrop8000 spectrophotometer. Total RNA integrity was confirmed using an Agilent Technologies 2100 Bioanalyzer with an RNA Integrity Number value. Total RNA sequencing libraries were prepared according to the manufacturer’s instructions (Illumina Truseq Stranded Total RNA Sample Prep kit with Ribo-zero human). Total RNA was subjected to ribosomal RNA depletion, with Ribo-zero human reagent, using biotinylated probes that selectively bind rRNA species. Following purification, the rRNA-depleted total RNA was fragmented into small pieces using divalent cations under elevated temperature. The cleaved RNA fragments were copied into first-strand cDNAs using reverse transcriptase and random primers. This was followed by second-strand cDNA synthesis using DNA polymerase I and RNase H. A single “A” base was added to the cDNA fragments, which were subsequently ligated with the adapter. The products were purified and enriched, using PCR to create the final cDNA library. The quality of the amplified libraries was checked by capillary electrophoresis (Bioanalyzer, Agilent). After QPCR using SYBR Green PCR Master Mix (Applied Biosystems), we pooled index-tagged libraries in equimolar amounts. Cluster generation occurred in the flow cell on the cBot automated cluster generation system (Illumina). Then, the flow cell was loaded into the Novaseq 6000 sequencing system (Illumina), and sequencing was conducted with a 2 × 100 bp read length.

### Statistical analysis

All experimental data are showed as the mean ± SEM. One-way repeated measure ANOVAs (RM ANOVA) (within-subject factor: treatment) were run, followed by Tukey’s or Scheffe post hoc tests to evaluate treatment effects. A *P* value of < 0.05 was regarded as significant. Statistical analyses were conducted using R software version 3.5.1.

## Results

### Pathologic changes in the lung tissue

**Fig. 1:**
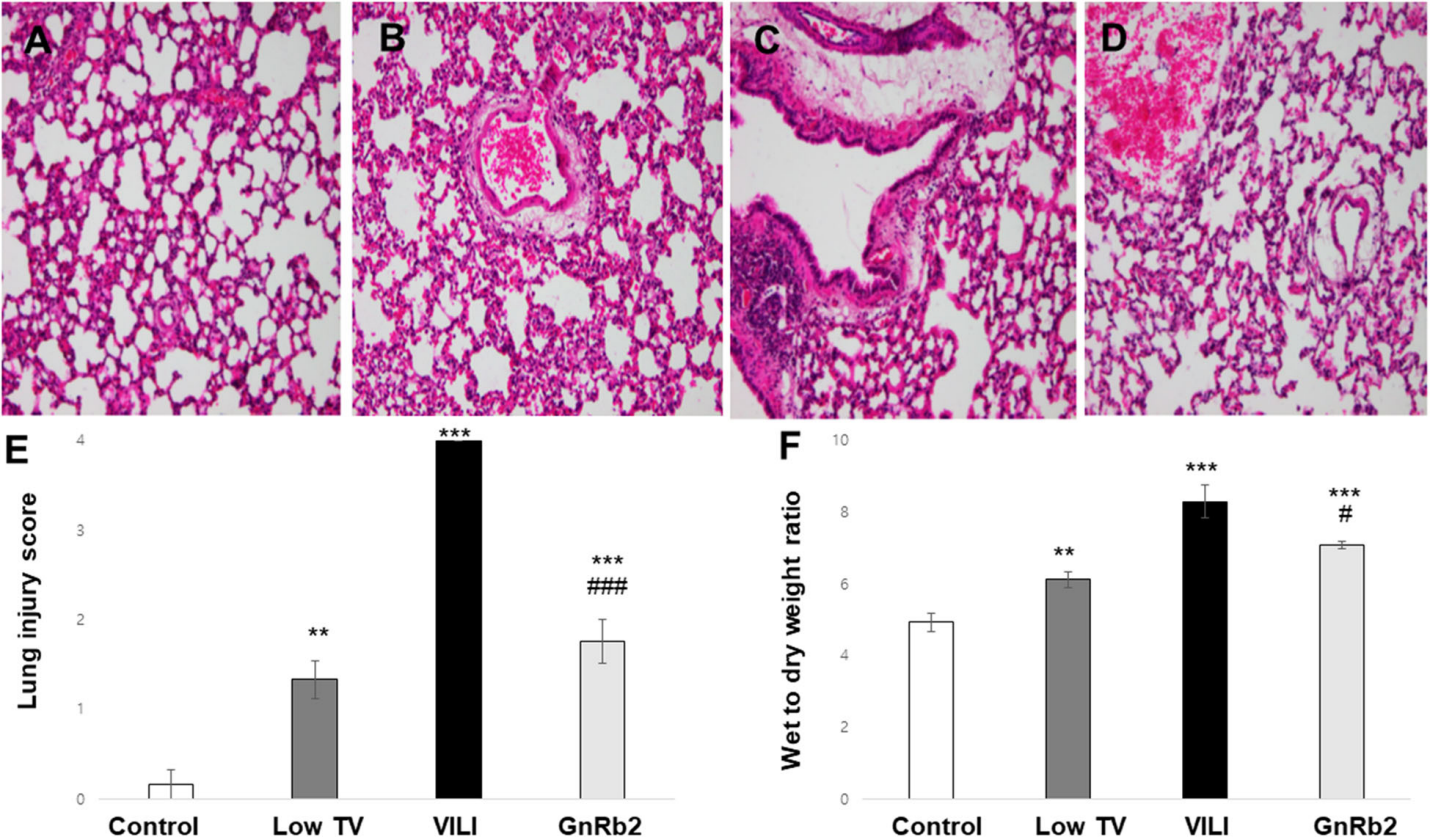
Pathologic findings in the lung tissue, lung injury scores, and pulmonary edema. Representative histological images of each group are shown. Tissue sections were stained with hematoxylin and eosin (× 200). **a** Normal lung structures are shown in the control group. **b** Mild neutrophils infiltration was observed in the low TV group. **c** Significant thickening of alveolar septal walls, neutrophil infiltration, and alveolar edema can be observed in the VILI group. **d** Significantly reduced degrees of lung injury, including decreased edema and inflammatory cell infiltration, can be observed in the GnRb2 group. **e** The lung injury scores were significantly higher in the low TV, VILI, and GnRb2 group compared to the control group, respectively (low TV; *p* < 0.01, VILI; *p* < 0.001, GnRb2; *p* < 0.001). GnRb2 pretreatment significantly suppressed the increased lung injury scores induced by VILI (*p* < 0.001). **f** Mechanical ventilation markedly increased the wet-to-dry weight ratio. Wet-to-dry ratio was significantly higher in the low TV, VILI, and GnRb2 group, respectively (low TV; *p* < 0.01, VILI; *p* < 0.001, GnRb2; *p* < 0.001). Pretreatment with GnRb2 significantly reduced wet-to-dry weight ratios compared to the VILI group (*p* = 0.048) (*n* = 6 in each group). **p < 0.01 compared to control, ***p < 0.001 compared to control, ^#^*p* < 0.05 compared to VILI, ^###^*p* < 0.001 compared to VILI

Histological examination of the lung tissues in the control group showed complete pulmonary alveoli structures, no alveolar edema, no significant inflammatory cell infiltration, and no evidence of hyaline membrane formation (Fig. 1a). The low TV group showed mild interstitial neutrophil infiltration (Fig. 1b). The VILI group showed interstitial perivascular edema, intra-alveolar and interstitial neutrophil infiltration, alveolar hemorrhage, and hyaline membrane formation (Fig. 1c). The lungs of the GnRb2 group presented with only minor signs of alveolar edema and inflammatory cell infiltration (Fig. 1d).

### Lung injury score

The lung injury scores were significantly higher in the low TV, VILI, and GnRb2 group compared to the control group, respectively (low TV; *p* < 0.01, VILI; *p* < 0.001, GnRb2; *p* < 0.001). GnRb2 pretreatment significantly attenuated the increased lung injury score induced by VILI (*p* < 0.001) (Fig. 1e).

### Assessment of pulmonary edema

Wet-to-dry ratio was significantly higher in the low TV, VILI, and GnRB2 group, respectively (low TV; *p* < 0.01, VILI; *p* < 0.001, GnRb2; *p* < 0.001). Wet-to-dry weight ratios were reduced in the GnRb2 group compared to the VILI group (*p* < 0.05) (Fig. 1f).

### Cytokine levels in BALF

**Fig. 2:**
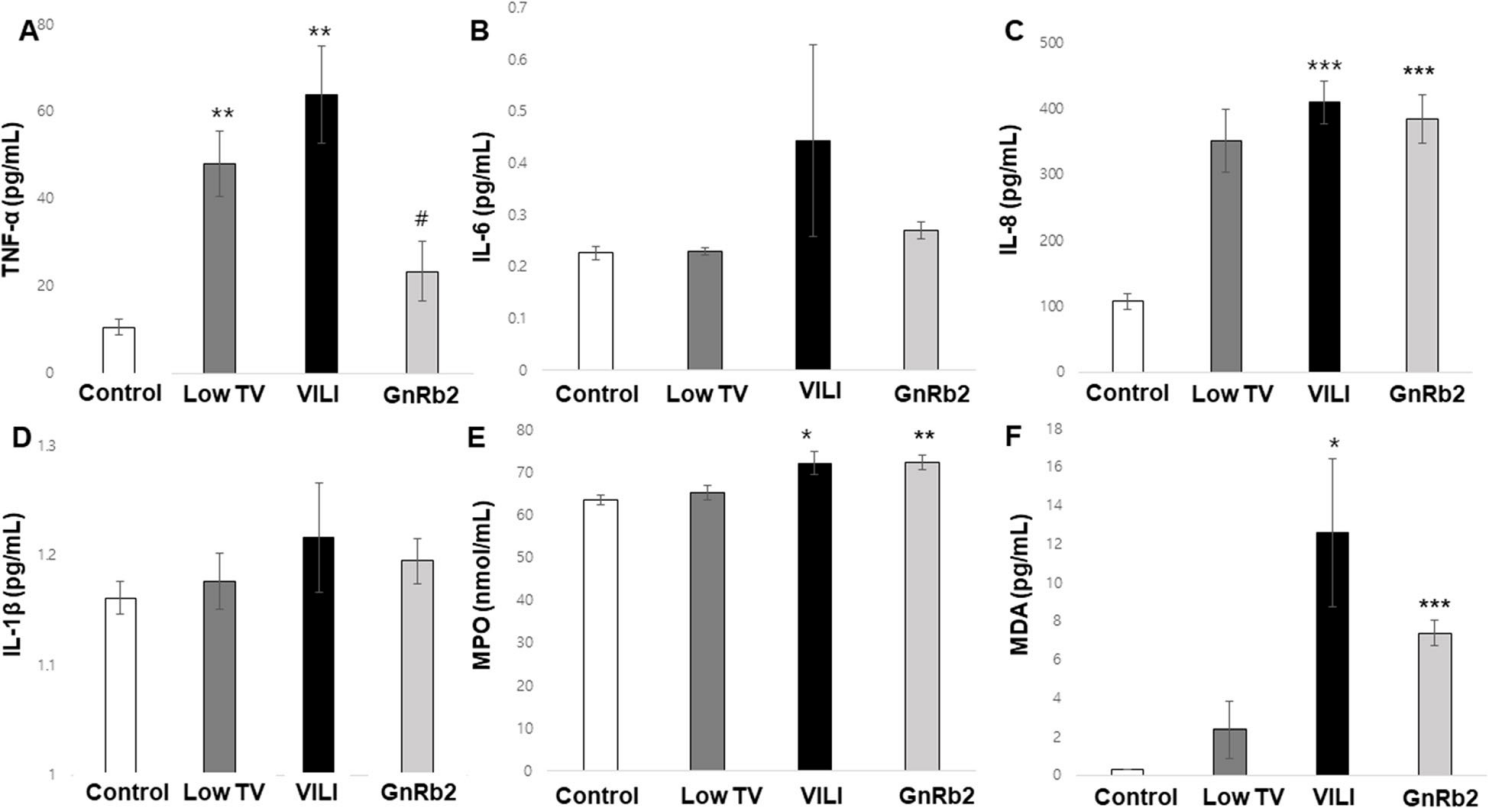
Cytokine levels and protein activity in BALF. **a** Mechanical ventilation markedly increased TNF-α concentration in BALF (low TV; *p* < 0.01, VILI; *p* < 0.01). Pretreatment with GnRb2 attenuated the increased TNF-α concentration induced by VILI (*p* < 0.05). **f** MDA concentration was markedly increased in the VILI group compared to the control group and tended to decrease in response to GnRb2 pretreatment compared to the VILI group. *p < 0.05 compared to control, **p < 0.01 compared to control, ***p < 0.001 compared to control, ^#^*p* < 0.05 compared to VILI (*n* = 6 in each group)

The TNF-α concentration in BALF was significantly higher in the low TV and VILI group compared to the control group (low TV; *p* = 0.004, VILI; *p* = 0.004). The TNF-α concentration in the GnRb2 group was decreased compared to the VILI group (*p* = 0.014) (Fig. 2a). IL-8 also increased significantly in the VILI compared to the control group (*p* < 0.001, Fig. 2c). However, IL-8 in the GnRb2 group did not significantly decrease compared to the VILI group. As well, IL-6 and IL-1β did not differ between the groups (Fig. 2b, d).

### The MPO activity in BALF

The BALF MPO activity was significantly higher in the VILI group compared to the control group (*p* = 0.011). But there was no significant difference in BALF MPO activity between the VILI and GnRb2 groups (Fig. 2e).

### Oxidative stress in the lung tissue

The MDA concentration in BALF was significantly increased in the VILI group compared to the control group (*p* = 0.024). MDA was decreased in the GnRb2 group compared to the VILI group, but it was not statistically significant (*p* = 0.234) (Fig. 2f).

### Differentially expressed genes

**Fig. 3:**
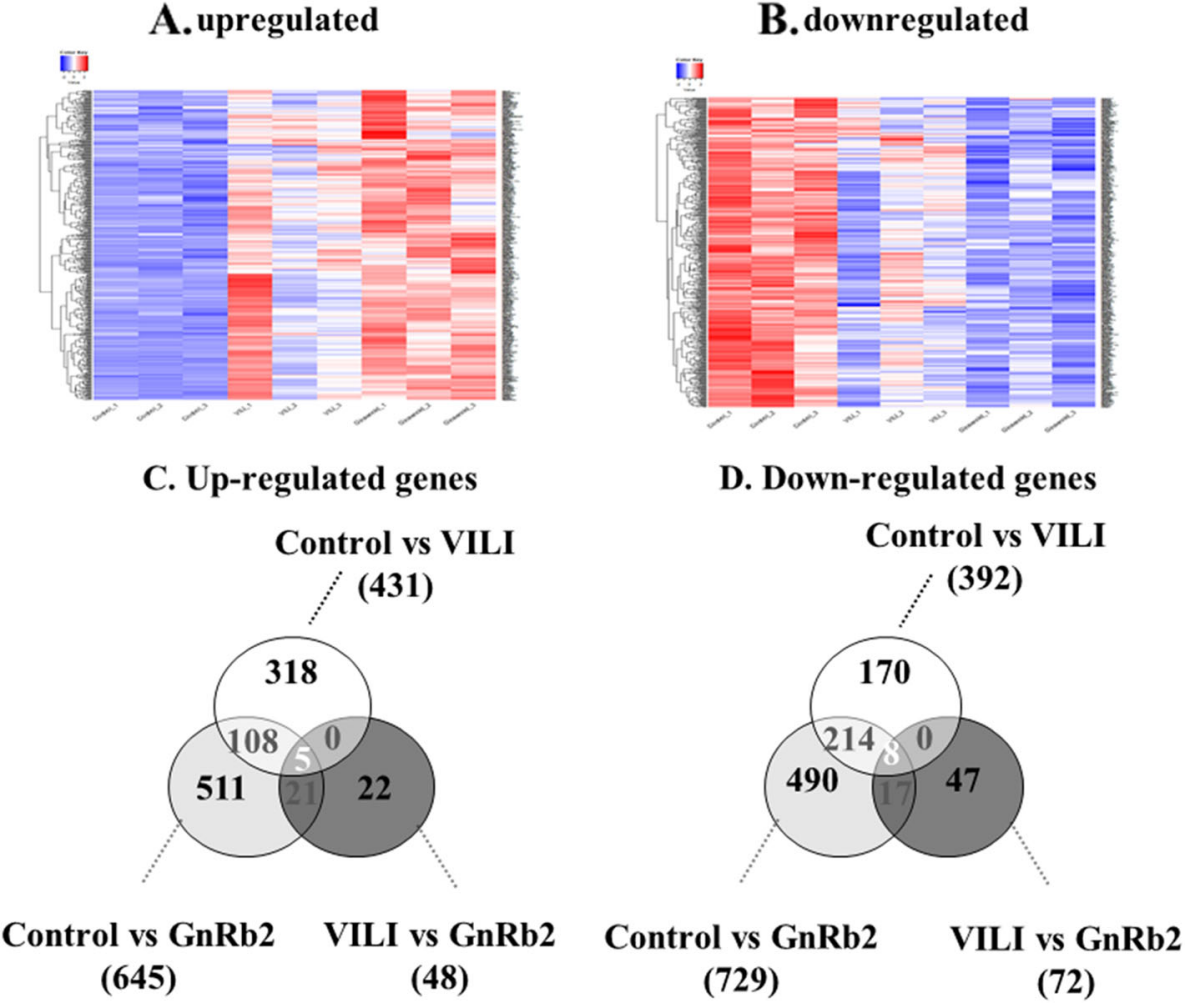
Comparative heatmap depiction of differential gene expression and differentially expressed genes between three groups. **a** Heatmap of upregulated genes. **b** Heatmap of downregulated genes. **c**, **d** The Venn diagram shows the number of overlapping altered genes between the control, VILI, and GnRb2 groups

**Fig. 4:**
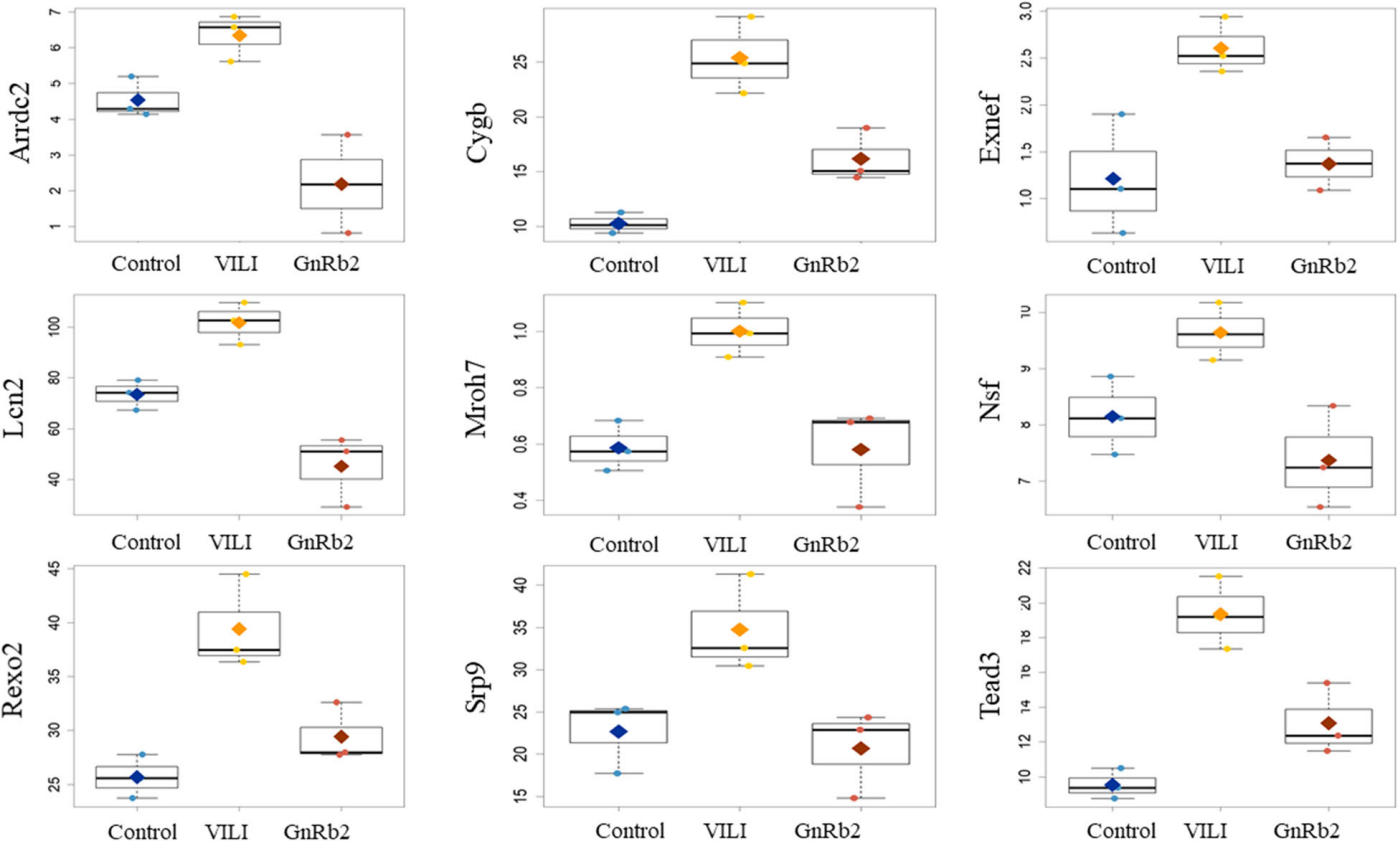
Upregulated gene expression profiles in the experimental groups. Genes that were significantly upregulated in the VILI group and significantly different in the GnRb2 group (*p* < 0.05) are shown. Of the 431 genes that were upregulated in the VILI group, only 9 genes (*Arrdc2*, *Cygb*, *Exnef*, *Lcn2*, *Mroh7*, *Nsf*, *Rexo2*, *Srp9*, and *Tead3*) recovered to control levels in the GnRb2 group

**Fig. 5:**
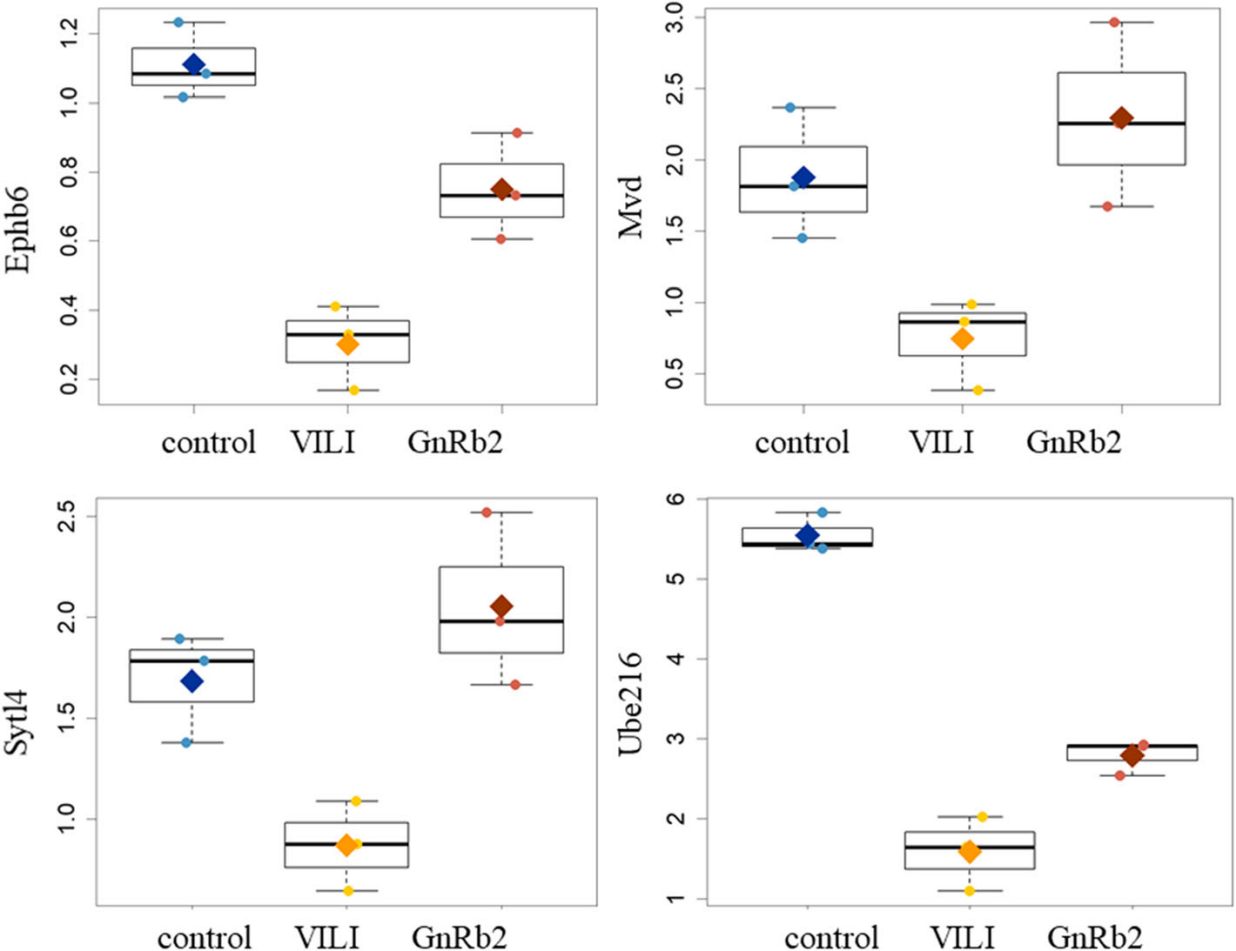
Downregulated gene expression profiles in the experimental groups. Genes that were significantly downregulated in the VILI group and significantly different in the GnRb2 group (*p* < 0.05) are shown. Of the 392 genes that were downregulated in the VILI group, only 4 genes (*Ephb6*, *Mvd*, *Sytl4*, and *Ube2l6*) recovered to control levels in the GnRb2 group

Results of the gene array experiments are presented as fold changes between the three groups: control, VILI, and GnRb2. Comparisons were performed step by step between the three groups (control vs VILI, VILI vs GnRb2). A heatmap of genes that differed significantly between the three groups is shown in Fig. 3 (a, upregulated genes; b, downregulated genes). We identified 823 differentially expressed genes in the VILI group compared to the control group, including 431 upregulated and 392 downregulated genes (Fig. 3c, d). The inflammatory cytokines such as TNF-α (*p* = 0.084), IL-1β (*p* = 0.352), and IL-18 (*p* = 0.725) were increased in the VILI group compared to the control, but not significant. Of the 431 genes that were upregulated in the VILI group, only 9 genes (*Arrdc2*, *Cygb*, *Exnef*, *Lcn2*, *Mroh7*, *Nsf*, *Rexo2*, *Srp9*, and *Tead3*) recovered to control levels in the GnRb2 group (Fig. 4). Of the 392 genes that were downregulated in the VILI, only 4 genes (*Ephb6*, *Mvd*, *Sytl4*, and *Ube2l6*) recovered to control levels in the GnRb2 group (Fig. 5).

### Immunohistochemical expression of TNF-α

**Fig. 6:**
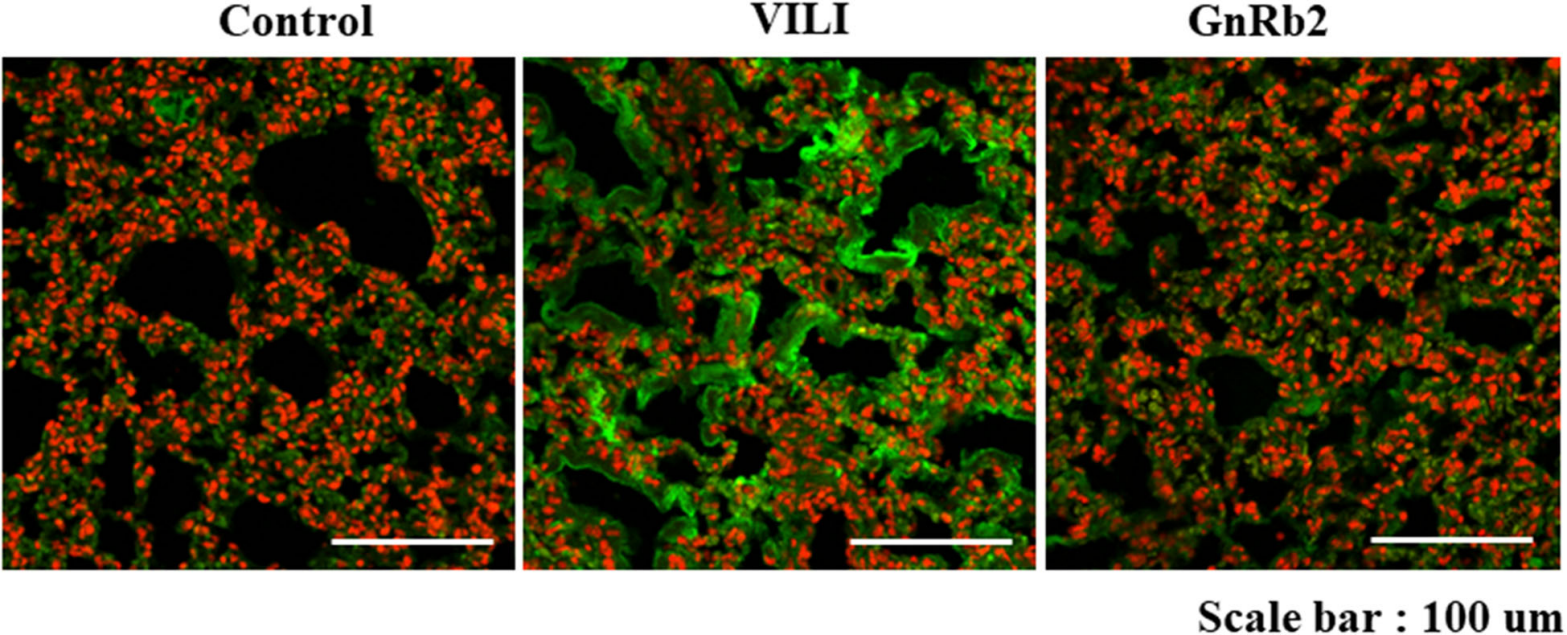
Representative images for the immunohistochemical staining for TNF-α in the three groups. TNF-α increased in the VILI group compared to the control group. In the GnRb2 group, TNF-α expression decreased compared to the VILI group

Representative images showing the immunohistochemical expression of TNF-α in the three groups are shown in Fig. 6. Immunohistochemical staining of TNF-α was more intense in the VILI group compared to the control group. Immunohistochemical staining for TNF-α was weaker in the GnRb2 group compared to the VILI group.

## Discussion

Our study shows the anti-inflammatory effects of GnRb2, which is commonly used as an herbal medicine, in an experimental rat model of VILI. Intraperitoneal injection of GnRb2 alleviated the biochemical and histologic changes induced by VILI. These anti-inflammatory effects of GnRb2 may be mediated via the suppression of TNF-α activation. Among the upregulated or downregulated genes in the VILI group, only 13 genes (*Arrdc2*, *Cygb*, *Exnef*, *Lcn2*, *Mroh7*, *Nsf*, *Rexo2*, *Srp9*, *Tead3*, *Ephb6*, *Mvd*, *Sytl4*, and *Ube2l6*) recovered to control levels in the GnRb2 group. Of the 13 genes, lipocalin 2 (LCN2) is a potential downstream target of TNF-α leading to the protective effect of GnRb2 in the VILI model.

The anti-inflammatory effects of Ginseng derivatives have been demonstrated in various experimental models, mainly cell studies [17, 18]. However, the pathogenic mechanisms of the anti-inflammatory effects are not fully understood and the effects of Ginseng derivatives in a VILI model have not been studied. In this study, pretreatment with GnRb2 decreased wet-to-dry weight ratio, MDA concentration, and histologic lung injury score in a rat model of VILI. These results support our hypothesis that GnRb2 protects the lung from VILI and prevents the progress of pulmonary edema; the typical pathological damage induced by VILI is mitigated by GnRb2.

In our study, GnRb2 pretreatment decreased TNF-α concentration in BALF and immunohistochemistry staining of TNF-α. Previously, GnRb2 has been shown to inhibit TNF-α production and/or inhibit the transcriptional activity of NF-kB, but the mechanism is not well understood [8, 9, 19]. Generally, NF-kB exists as inactive NF-kB dimers in the cytosol of unstimulated cells and is activated by phosphorylation of the inhibitor of NF-kB proteins (IkBs) [20]. Various cellular stimuli such as TNF-α lead to degradation of IkBs via IκB kinase (IKK) complex, which is responsible for IκB phosphorylation. Previously, Wu et al. reported that GnRb2 inhibits TNF-α production through NF-kB inhibition by inhibition of phosphorylation of IkBα [21]. Unfortunately, gene expression profiling of this study did not find any difference in the level of IkBs and IKK among the three groups. As well, there were no significant differences in gene expression profiling of the promoter genes of TNF-α between the three groups. Only a significant change in the LCN2 gene, one of the downstream changes of TNF-α, was observed, suggesting a potential role of ginsenosides through the TNF-α-LCN2 pathway. LCN2 is derived from specific neutrophil granules and is widely participated in inflammatory and immune responses as a marker of neutrophil activation [22]. In a previous study, LCN2 expression increased in VILI animal models in accordance with the degree of lung injury [23]. LCN2 activation can be mediated by pro-inflammatory cytokines, such as IL-1β and TNF-α [24–26]. In this study, we failed to reveal whether the GnRb2 affect the upregulation of LCN2 through NF-kB or an alternative pathway. Given that there is no significant difference in the level of NF-kB among gene expression profiling between the three groups, there is a possibility of the upregulation of LCN2 through an alternative pathway. Further researches are required to verify a potential alternative pathway suppressing the level of TNF-α by GnRb2 in a VILI model.

To date, of the remaining 12 genes, there are no reports associated with VILI. All of 13 genes are potentially associated with crosstalk between VILI and the therapeutic effect of GnRb2. However, we could not find the additional biochemical evidence to convince the rest of the 12 genes except LCN2 were related to VILI and GnRb2. N-ethylmaleimide sensitive factor (NSF) is related to the secretion of inflammatory mediators from pulmonary microvascular endothelial cells and vascular instability [27]. Mechanical stress in the pulmonary vasculature is one of the mechanisms causing pathophysiologic changes in VILI [28]. In this study, GnRb2 reduces NSF expression in VILI, suggesting that the protective effect of GnRb2 might be associated with the endothelial injury. Further research is required on other genes, including NSF.

This study has several limitations including a small sample size and uncertainty about the fidelity of FFPE samples. First, there are concerns regarding data quality and interpretation of RNA derived from FFPE. FFPE tissue processing and sample storage are known to result in highly degraded RNA, which may limit gene detection and introduces sequencing artifacts. However, as recent technical advances, extracting RNA from FFPE tissues does not affect the overall quality of results [29–33]. The variable reproducibility of gene signatures probably relates to the reliability of the individual gene selected and possibly to the algorithm. Second, this study did not fully confirm the cellular response in protein level, only found the significant changes of TNF-α through the ELISA test and IHC stain. Although this is partially convincing evidence, GnRb2 was associated with TNF-α reduction and its downstream, LCN2. Based on these pilot results, further studies are required to verify the therapeutic mechanism of GnRb2 on TNF-α level and the significance of the 13 genes in a VILI and GnRb2 treatment. Third, the arterial oxygen tension was not measured in our VILI model. Therefore, this finding should be cautiously interpreted to apply real clinical practice due to a lack of physiologic benefits regarding oxygenation improvement. Further scientific evidence aiming at physiologic benefit should be preceded to a future clinical trial.

## Conclusions

In conclusion, our study showed that the GnRb2 pretreatment attenuates VILI, including inflammation and pulmonary edema. Thirteen genes were differentially expressed in response to GnRb2 pretreatment in VILI. Among the differentially expressed genes, the effect of GnRb2 on lung injury is associated with the downregulation of LCN2 by TNF-α inhibition. GnRb2 is a potential therapeutic agent for treating or preventing VILI through inhibition of the TNF-α signaling pathway.

## Supplementary Information

**Additional file 1:.** Supplement 1. ELISA kits used in this study.

## Data Availability

The datasets used and/or analyzed during the current study are available from the corresponding author on reasonable request.

## Abbreviations

ALI: Acute lung injury
ARDS: Acute respiratory distress syndrome
VILI: Ventilator-induced lung injury
GnRb2: Ginsenoside Rb2
MV: Mechanical ventilation
BALF: Bronchoalveolar lavage fluid
MPO: Myeloperoxidase
MDA: Malondialdehyde
LCN2: Lipocalin 2
