# Supplementary material for: Ginsenoside ameliorated ventilator-induced lung injury in rats
Source: J Intensive Care. 2020 Nov 23;8:89. doi: 10.1186/s40560-020-00509-5 (PMC7682776; doi:10.1186/s40560-020-00509-5)
Supplement: Supplementary file 1 — Additional file 1:. Supplement 1. ELISA kits used in this study. [file 40560_2020_509_MOESM1_ESM.docx]

Supplement 1. ELISA kits used in this study

| ELISA kit | Source |
| --- | --- |
| MPO kit | ab105136, Abcam, UK |
| MDA kit | ab118970, Abcam, UK |
| rat IL-6 ELISA kit | ab100772, Abcam, UK |
| rat GRO/CINC-1 assay kit | 27162, Immuno-Biological Laboratories, Japan |
| IL-1β Rat ELISA kit | ab100768, Abcam, UK |
| TNF-α Rat ELISA kit | ab46070, Abcam, UK |
